# Supplementary material for: Hot spot prediction in protein-protein interactions by an ensemble system
Source: BMC Syst Biol. 2018 Dec 31;12(Suppl 9):132. doi: 10.1186/s12918-018-0665-8 (PMC6311905; doi:10.1186/s12918-018-0665-8)
Supplement: Supplementary file 2 — All relevant MATLAB code. A simple Matlab implement of our predictor. (ZIP 19,742 kb) [file 12918_2018_665_MOESM2_ESM.zip › Additional file 2/Read ASA/ASA website/dbMPIKT/4NZWB.html]

 NetSurfP - Protein Surface Accessibility and Secondary Structure Predictions

  

|  |  |  |
| --- | --- | --- |
|  |  | NetSurfP - Protein Surface Accessibility and Secondary Structure Predictions  Technical University of Denmark |

  


---

```
# For publication of results, please cite:
# A generic method for assignment of reliability scores applied to solvent accessibility predictions.
# Bent Petersen, Thomas Nordahl Petersen, Pernille Andersen, Morten Nielsen and Claus Lundegaard
# BMC Structural Biology 2009, 9:51 doi:10.1186/1472-6807-9-51
#
# Column 1: Class assignment - B for buried or E for Exposed - Threshold: 25% exposure, but not based on RSA
# Column 2: Amino acid
# Column 3: Sequence name
# Column 4: Amino acid number
# Column 5: Relative Surface Accessibility - RSA
# Column 6: Absolute Surface Accessibility
# Column 7: Z-fit score for RSA prediction
# Column 8: Probability for Alpha-Helix
# Column 9: Probability for Beta-strand
# Column 10: Probability for Coil
E Q  4NZWB                  1    0.832 148.559   1.235   0.246   0.004   0.750
E A  4NZWB                  2    0.523  57.613  -1.008   0.717   0.014   0.269
E M  4NZWB                  3    0.374  74.917  -0.280   0.717   0.014   0.269
E A  4NZWB                  4    0.596  65.657   0.192   0.717   0.014   0.269
E H  4NZWB                  5    0.461  83.929   0.691   0.622   0.015   0.363
B L  4NZWB                  6    0.207  37.957  -0.760   0.522   0.016   0.462
E R  4NZWB                  7    0.550 125.904   0.141   0.430   0.016   0.555
E G  4NZWB                  8    0.556  43.781  -0.075   0.339   0.016   0.645
B F  4NZWB                  9    0.148  29.784  -0.132   0.339   0.016   0.645
E A  4NZWB                 10    0.381  41.931  -1.336   0.257   0.016   0.727
E N  4NZWB                 11    0.565  82.701  -1.134   0.257   0.016   0.727
E Q  4NZWB                 12    0.473  84.549  -0.909   0.181   0.016   0.803
E H  4NZWB                 13    0.589 107.103  -2.262   0.115   0.016   0.868
E S  4NZWB                 14    0.418  49.036  -1.341   0.058   0.017   0.925
E R  4NZWB                 15    0.555 127.118  -1.141   0.058   0.017   0.925
E V  4NZWB                 16    0.577  88.716  -0.241   0.058   0.017   0.925
E D  4NZWB                 17    0.413  59.513  -0.160   0.016   0.005   0.979
B P  4NZWB                 18    0.162  23.059  -0.406   0.600   0.003   0.397
E E  4NZWB                 19    0.537  93.831   0.592   0.782   0.003   0.216
E E  4NZWB                 20    0.617 107.755   0.873   0.782   0.003   0.216
E L  4NZWB                 21    0.296  54.143   1.361   0.802   0.014   0.185
B F  4NZWB                 22    0.069  13.748   0.739   0.831   0.044   0.125
E T  4NZWB                 23    0.564  78.296   1.453   0.779   0.100   0.120
E K  4NZWB                 24    0.383  78.783   1.285   0.725   0.163   0.112
B L  4NZWB                 25    0.171  31.237   0.579   0.605   0.105   0.290
E D  4NZWB                 26    0.457  65.868   0.964   0.428   0.171   0.402
E R  4NZWB                 27    0.288  66.066   1.338   0.321   0.252   0.427
B I  4NZWB                 28    0.062  11.489   0.447   0.135   0.317   0.548
B G  4NZWB                 29    0.182  14.300  -1.181   0.064   0.216   0.721
E K  4NZWB                 30    0.544 111.921   0.495   0.019   0.141   0.840
B G  4NZWB                 31    0.250  19.691  -2.477   0.018   0.088   0.893
B S  4NZWB                 32    0.188  22.022  -1.361   0.018   0.088   0.893
E F  4NZWB                 33    0.310  62.237  -0.934   0.021   0.279   0.699
B G  4NZWB                 34    0.063   4.958  -0.730   0.023   0.655   0.322
B E  4NZWB                 35    0.227  39.622   1.353   0.011   0.918   0.071
B V  4NZWB                 36    0.027   4.104   0.697   0.006   0.962   0.032
B Y  4NZWB                 37    0.122  25.965   1.315   0.000   0.983   0.017
B K  4NZWB                 38    0.135  27.811   1.561   0.000   0.983   0.017
B G  4NZWB                 39    0.017   1.369   0.499   0.000   0.983   0.017
B I  4NZWB                 40    0.207  38.202   1.664   0.000   0.983   0.017
B D  4NZWB                 41    0.175  25.145   0.444   0.001   0.959   0.040
E N  4NZWB                 42    0.263  38.474   0.889   0.004   0.514   0.481
E H  4NZWB                 43    0.684 124.456  -0.079   0.004   0.197   0.799
E T  4NZWB                 44    0.589  81.639   0.244   0.005   0.045   0.951
E K  4NZWB                 45    0.525 108.034   0.090   0.005   0.015   0.979
E E  4NZWB                 46    0.347  60.656   1.604   0.004   0.514   0.481
B V  4NZWB                 47    0.193  29.618   1.734   0.001   0.959   0.040
B V  4NZWB                 48    0.052   7.931   1.854   0.001   0.959   0.040
B A  4NZWB                 49    0.019   2.149   0.946   0.001   0.959   0.040
B I  4NZWB                 50    0.047   8.769   0.760   0.000   0.983   0.017
B K  4NZWB                 51    0.083  17.011   0.420   0.000   0.983   0.017
B I  4NZWB                 52    0.193  35.779   1.178   0.001   0.959   0.040
B I  4NZWB                 53    0.064  11.822   0.871   0.001   0.959   0.040
E D  4NZWB                 54    0.477  68.736   1.512   0.002   0.816   0.182
B L  4NZWB                 55    0.106  19.482   0.285   0.005   0.262   0.733
E E  4NZWB                 56    0.725 126.692   0.918   0.005   0.045   0.951
E E  4NZWB                 57    0.644 112.542   0.576   0.003   0.003   0.994
E A  4NZWB                 58    0.425  46.824   0.218   0.016   0.005   0.979
E E  4NZWB                 59    0.692 120.857   0.590   0.858   0.002   0.139
E D  4NZWB                 60    0.688  99.170   1.409   0.923   0.002   0.076
E E  4NZWB                 61    0.309  53.965   1.176   0.923   0.002   0.076
B I  4NZWB                 62    0.177  32.671   0.850   0.970   0.001   0.030
E E  4NZWB                 63    0.595 103.947   1.369   0.970   0.001   0.030
E D  4NZWB                 64    0.411  59.240   2.095   0.970   0.001   0.030
B I  4NZWB                 65    0.021   3.848   1.049   0.970   0.001   0.030
E Q  4NZWB                 66    0.296  52.866   1.298   0.970   0.001   0.030
E Q  4NZWB                 67    0.439  78.334   1.551   0.970   0.001   0.030
B E  4NZWB                 68    0.058  10.185   0.117   0.970   0.001   0.030
B I  4NZWB                 69    0.052   9.620   0.710   0.988   0.000   0.012
E T  4NZWB                 70    0.472  65.397   1.725   0.988   0.000   0.012
B V  4NZWB                 71    0.097  14.924   0.850   0.970   0.001   0.030
B L  4NZWB                 72    0.083  15.124   0.159   0.970   0.001   0.030
E S  4NZWB                 73    0.451  52.810   0.671   0.858   0.002   0.139
E Q  4NZWB                 74    0.552  98.516   1.063   0.600   0.003   0.397
B C  4NZWB                 75    0.074  10.333   0.863   0.176   0.004   0.820
E D  4NZWB                 76    0.687  99.026   0.614   0.016   0.005   0.979
B S  4NZWB                 77    0.171  20.006   0.081   0.003   0.003   0.994
E P  4NZWB                 78    0.609  86.460   1.053   0.018   0.019   0.964
B Y  4NZWB                 79    0.226  48.360  -0.226   0.052   0.084   0.864
B I  4NZWB                 80    0.040   7.419   0.737   0.021   0.451   0.528
B T  4NZWB                 81    0.056   7.726   0.736   0.021   0.756   0.223
E R  4NZWB                 82    0.317  72.685   1.048   0.018   0.846   0.136
B Y  4NZWB                 83    0.052  11.112   0.616   0.018   0.846   0.136
B F  4NZWB                 84    0.185  37.210   1.090   0.018   0.846   0.136
B G  4NZWB                 85    0.124   9.790  -2.063   0.002   0.816   0.182
B S  4NZWB                 86    0.080   9.423  -0.005   0.001   0.900   0.099
B Y  4NZWB                 87    0.166  35.474  -0.122   0.001   0.959   0.040
E L  4NZWB                 88    0.342  62.547   0.558   0.001   0.959   0.040
E K  4NZWB                 89    0.432  88.760   0.604   0.003   0.718   0.279
E S  4NZWB                 90    0.720  84.361   0.364   0.005   0.045   0.951
E T  4NZWB                 91    0.530  73.553  -0.006   0.005   0.015   0.979
B K  4NZWB                 92    0.236  48.483   1.821   0.002   0.816   0.182
B L  4NZWB                 93    0.026   4.706   1.038   0.001   0.959   0.040
B W  4NZWB                 94    0.061  14.767   1.357   0.006   0.962   0.032
B I  4NZWB                 95    0.020   3.700   1.612   0.001   0.959   0.040
B I  4NZWB                 96    0.020   3.681   1.349   0.001   0.959   0.040
B M  4NZWB                 97    0.024   4.762   1.335   0.011   0.918   0.071
B E  4NZWB                 98    0.055   9.696   0.317   0.002   0.816   0.182
B Y  4NZWB                 99    0.084  17.929   0.215   0.004   0.616   0.381
B L  4NZWB                100    0.040   7.287   0.002   0.019   0.141   0.840
E G  4NZWB                101    0.478  37.626  -0.135   0.005   0.015   0.979
E G  4NZWB                102    0.354  27.876  -1.637   0.058   0.017   0.925
B G  4NZWB                103    0.209  16.433  -1.334   0.115   0.016   0.868
B S  4NZWB                104    0.205  23.979  -0.152   0.430   0.016   0.555
B A  4NZWB                105    0.037   4.033  -0.353   0.923   0.002   0.076
E L  4NZWB                106    0.304  55.699   0.902   0.923   0.002   0.076
E D  4NZWB                107    0.406  58.562   1.261   0.923   0.002   0.076
B L  4NZWB                108    0.098  17.962   1.038   0.923   0.002   0.076
B L  4NZWB                109    0.079  14.483   0.143   0.858   0.002   0.139
E K  4NZWB                110    0.571 117.372   1.633   0.694   0.003   0.303
E P  4NZWB                111    0.658  93.313   0.767   0.176   0.004   0.820
E G  4NZWB                112    0.511  40.239   0.221   0.016   0.005   0.979
E P  4NZWB                113    0.583  82.671   0.436   0.005   0.015   0.979
B L  4NZWB                114    0.053   9.631  -0.300   0.003   0.003   0.994
E E  4NZWB                115    0.562  98.146   1.336   0.003   0.003   0.994
B E  4NZWB                116    0.128  22.414  -0.513   0.923   0.002   0.076
E T  4NZWB                117    0.596  82.721   0.696   0.970   0.001   0.030
B Y  4NZWB                118    0.174  37.248   1.657   0.970   0.001   0.030
B I  4NZWB                119    0.015   2.830   1.386   0.970   0.001   0.030
B A  4NZWB                120    0.077   8.463   1.262   0.970   0.001   0.030
E T  4NZWB                121    0.329  45.660   1.806   0.988   0.000   0.012
B I  4NZWB                122    0.053   9.805   1.504   0.970   0.001   0.030
B L  4NZWB                123    0.018   3.259   1.410   0.988   0.000   0.012
B R  4NZWB                124    0.231  52.830   1.596   0.988   0.000   0.012
B E  4NZWB                125    0.147  25.733   0.444   0.988   0.000   0.012
B I  4NZWB                126    0.026   4.847   1.246   0.988   0.000   0.012
B L  4NZWB                127    0.033   6.097  -0.007   0.988   0.000   0.012
E K  4NZWB                128    0.287  58.933   1.221   0.970   0.001   0.030
B G  4NZWB                129    0.034   2.699  -0.829   0.970   0.001   0.030
B L  4NZWB                130    0.034   6.244   0.285   0.970   0.001   0.030
E D  4NZWB                131    0.327  47.193   1.019   0.970   0.001   0.030
B Y  4NZWB                132    0.202  43.210   0.650   0.970   0.001   0.030
B L  4NZWB                133    0.045   8.166   0.851   0.938   0.007   0.055
B H  4NZWB                134    0.198  36.107   0.703   0.782   0.003   0.216
E S  4NZWB                135    0.743  87.080   1.007   0.406   0.004   0.590
E E  4NZWB                136    0.546  95.369   1.228   0.016   0.005   0.979
E R  4NZWB                137    0.533 122.034   0.056   0.005   0.015   0.979
B K  4NZWB                138    0.179  36.820   1.420   0.004   0.197   0.799
B I  4NZWB                139    0.108  20.036   0.498   0.022   0.552   0.426
B H  4NZWB                140    0.150  27.230   0.743   0.022   0.552   0.426
B R  4NZWB                141    0.233  53.265   0.233   0.066   0.296   0.638
B D  4NZWB                142    0.183  26.385  -0.518   0.056   0.142   0.802
B I  4NZWB                143    0.072  13.357   0.790   0.052   0.084   0.864
E K  4NZWB                144    0.316  64.898   0.924   0.053   0.043   0.903
B A  4NZWB                145    0.106  11.692  -0.863   0.053   0.043   0.903
E A  4NZWB                146    0.359  39.573  -0.136   0.018   0.047   0.935
B N  4NZWB                147    0.105  15.313  -0.738   0.064   0.216   0.721
B V  4NZWB                148    0.057   8.699   0.656   0.023   0.655   0.322
B L  4NZWB                149    0.073  13.293  -0.136   0.021   0.756   0.223
B L  4NZWB                150    0.081  14.776   0.694   0.023   0.655   0.322
B S  4NZWB                151    0.133  15.599   0.017   0.020   0.205   0.775
E E  4NZWB                152    0.603 105.344   0.094   0.018   0.047   0.935
E Q  4NZWB                153    0.711 126.985   0.653   0.005   0.015   0.979
B G  4NZWB                154    0.179  14.111  -0.303   0.018   0.019   0.964
E D  4NZWB                155    0.359  51.732   1.310   0.021   0.279   0.699
B V  4NZWB                156    0.034   5.210   0.436   0.018   0.846   0.136
B K  4NZWB                157    0.135  27.790   1.536   0.011   0.918   0.071
B L  4NZWB                158    0.034   6.262   0.472   0.011   0.918   0.071
B A  4NZWB                159    0.036   3.989  -0.162   0.018   0.846   0.136
B A  4NZWB                160    0.076   8.397  -1.410   0.079   0.592   0.329
B F  4NZWB                161    0.074  14.932  -0.006   0.216   0.235   0.548
B G  4NZWB                162    0.088   6.957  -0.983   0.453   0.248   0.299
B V  4NZWB                163    0.084  12.926   0.152   0.578   0.229   0.194
B A  4NZWB                164    0.033   3.615  -0.630   0.662   0.225   0.113
B G  4NZWB                165    0.217  17.086   0.491   0.649   0.163   0.188
B Q  4NZWB                166    0.290  51.705   0.824   0.594   0.285   0.121
B L  4NZWB                167    0.075  13.787   0.109   0.578   0.229   0.194
E T  4NZWB                168    0.367  50.889   0.155   0.502   0.102   0.396
E D  4NZWB                169    0.601  86.575  -0.003   0.268   0.043   0.689
E T  4NZWB                170    0.544  75.411  -0.600   0.268   0.043   0.689
E Q  4NZWB                171    0.590 105.285  -1.718   0.257   0.016   0.727
E I  4NZWB                172    0.512  94.739  -0.609   0.115   0.016   0.868
E K  4NZWB                173    0.425  87.525  -0.030   0.053   0.043   0.903
E R  4NZWB                174    0.295  67.532  -0.171   0.018   0.088   0.893
E N  4NZWB                175    0.387  56.701   0.429   0.019   0.141   0.840
B T  4NZWB                176    0.062   8.627  -0.355   0.021   0.451   0.528
B F  4NZWB                177    0.088  17.621  -0.417   0.021   0.756   0.223
B V  4NZWB                178    0.076  11.666  -0.464   0.021   0.756   0.223
B G  4NZWB                179    0.044   3.455  -1.622   0.022   0.552   0.426
B T  4NZWB                180    0.044   6.144   0.534   0.004   0.138   0.858
B P  4NZWB                181    0.069   9.777  -0.136   0.019   0.141   0.840
B F  4NZWB                182    0.094  18.786  -0.471   0.056   0.142   0.802
B W  4NZWB                183    0.066  15.921  -0.094   0.066   0.296   0.638
B M  4NZWB                184    0.038   7.684  -0.303   0.125   0.227   0.648
B A  4NZWB                185    0.039   4.309   0.127   0.268   0.043   0.689
B P  4NZWB                186    0.055   7.804  -0.050   0.561   0.047   0.393
B E  4NZWB                187    0.091  15.880  -0.239   0.578   0.229   0.194
B V  4NZWB                188    0.140  21.472   0.646   0.478   0.309   0.214
B I  4NZWB                189    0.075  13.967   0.466   0.478   0.309   0.214
E K  4NZWB                190    0.548 112.641   1.119   0.307   0.165   0.527
E Q  4NZWB                191    0.753 134.522  -0.333   0.053   0.043   0.903
E S  4NZWB                192    0.539  63.159   0.524   0.018   0.019   0.964
E A  4NZWB                193    0.610  67.200  -1.172   0.018   0.019   0.964
B Y  4NZWB                194    0.193  41.201  -0.166   0.018   0.047   0.935
E D  4NZWB                195    0.516  74.327  -0.271   0.018   0.019   0.964
E F  4NZWB                196    0.336  67.375   0.308   0.053   0.043   0.903
E K  4NZWB                197    0.356  73.311   0.821   0.191   0.086   0.723
B A  4NZWB                198    0.031   3.438   0.706   0.278   0.093   0.628
B D  4NZWB                199    0.060   8.588   0.479   0.455   0.046   0.498
B I  4NZWB                200    0.028   5.198   0.831   0.701   0.107   0.192
B W  4NZWB                201    0.049  11.736   0.083   0.831   0.044   0.125
B S  4NZWB                202    0.034   3.997  -0.992   0.879   0.010   0.111
B L  4NZWB                203    0.041   7.470   0.042   0.879   0.010   0.111
B G  4NZWB                204    0.038   3.030  -0.600   0.879   0.010   0.111
B I  4NZWB                205    0.026   4.884   0.749   0.938   0.007   0.055
B T  4NZWB                206    0.038   5.284   0.402   0.975   0.003   0.022
B A  4NZWB                207    0.019   2.083   1.315   0.970   0.001   0.030
B I  4NZWB                208    0.049   9.121   1.356   0.988   0.000   0.012
B E  4NZWB                209    0.033   5.835   0.077   0.970   0.001   0.030
B L  4NZWB                210    0.034   6.262   0.360   0.970   0.001   0.030
B A  4NZWB                211    0.040   4.419   0.368   0.858   0.002   0.139
B K  4NZWB                212    0.226  46.488  -0.215   0.502   0.002   0.495
E G  4NZWB                213    0.281  22.083  -0.697   0.109   0.005   0.886
E E  4NZWB                214    0.343  59.940   0.594   0.016   0.005   0.979
B P  4NZWB                215    0.251  35.560   0.360   0.016   0.005   0.979
B P  4NZWB                216    0.101  14.360  -1.499   0.003   0.003   0.994
B N  4NZWB                217    0.113  16.499  -0.201   0.003   0.003   0.994
E S  4NZWB                218    0.481  56.373  -0.222   0.003   0.003   0.994
E D  4NZWB                219    0.751 108.176  -0.696   0.003   0.003   0.994
E L  4NZWB                220    0.288  52.678  -0.096   0.053   0.005   0.942
E H  4NZWB                221    0.500  90.932   0.081   0.016   0.005   0.979
B P  4NZWB                222    0.156  22.179  -0.558   0.923   0.002   0.076
B M  4NZWB                223    0.243  48.644  -0.013   0.970   0.001   0.030
E R  4NZWB                224    0.386  88.279   1.553   0.970   0.001   0.030
B V  4NZWB                225    0.028   4.242   0.314   0.970   0.001   0.030
B L  4NZWB                226    0.046   8.496   0.389   0.970   0.001   0.030
B F  4NZWB                227    0.246  49.372   0.682   0.970   0.001   0.030
B L  4NZWB                228    0.233  42.699   1.346   0.970   0.001   0.030
B I  4NZWB                229    0.032   5.865   0.195   0.923   0.002   0.076
B P  4NZWB                230    0.235  33.318  -0.048   0.694   0.003   0.303
E K  4NZWB                231    0.672 138.148   0.287   0.321   0.003   0.675
E N  4NZWB                232    0.621  90.988  -0.558   0.053   0.005   0.942
E S  4NZWB                233    0.673  78.876  -0.333   0.016   0.005   0.979
E P  4NZWB                234    0.307  43.606  -0.310   0.003   0.003   0.994
E P  4NZWB                235    0.308  43.776  -0.496   0.003   0.003   0.994
E T  4NZWB                236    0.654  90.765   0.591   0.003   0.003   0.994
B L  4NZWB                237    0.086  15.692  -0.390   0.003   0.003   0.994
E E  4NZWB                238    0.370  64.622   0.507   0.016   0.005   0.979
E G  4NZWB                239    0.841  66.179  -0.237   0.246   0.004   0.750
E Q  4NZWB                240    0.549  98.034   1.064   0.321   0.003   0.675
B H  4NZWB                241    0.070  12.678   0.805   0.321   0.003   0.675
B S  4NZWB                242    0.094  10.958  -0.166   0.181   0.016   0.803
E K  4NZWB                243    0.695 142.900   1.093   0.923   0.002   0.076
E P  4NZWB                244    0.358  50.857   1.219   0.970   0.001   0.030
B F  4NZWB                245    0.018   3.512   1.148   0.970   0.001   0.030
E K  4NZWB                246    0.292  60.044   1.689   0.970   0.001   0.030
E E  4NZWB                247    0.485  84.799   1.546   0.970   0.001   0.030
B F  4NZWB                248    0.050   9.955   1.228   0.970   0.001   0.030
B V  4NZWB                249    0.023   3.597   1.022   0.970   0.001   0.030
E E  4NZWB                250    0.349  60.970   1.835   0.970   0.001   0.030
E A  4NZWB                251    0.363  39.981   2.064   0.970   0.001   0.030
B C  4NZWB                252    0.029   4.128   0.480   0.923   0.002   0.076
B L  4NZWB                253    0.059  10.894   0.571   0.782   0.003   0.216
E N  4NZWB                254    0.493  72.146   1.439   0.600   0.003   0.397
E K  4NZWB                255    0.419  86.106   1.388   0.321   0.003   0.675
E D  4NZWB                256    0.262  37.725   0.832   0.016   0.005   0.979
B P  4NZWB                257    0.208  29.515  -0.080   0.053   0.005   0.942
E R  4NZWB                258    0.769 176.032   0.924   0.109   0.005   0.886
E F  4NZWB                259    0.493  98.865   0.786   0.058   0.017   0.925
B R  4NZWB                260    0.216  49.350   0.013   0.018   0.019   0.964
B P  4NZWB                261    0.217  30.750  -0.268   0.058   0.017   0.925
E T  4NZWB                262    0.307  42.595   1.322   0.058   0.017   0.925
B A  4NZWB                263    0.036   3.967  -0.412   0.923   0.002   0.076
E K  4NZWB                264    0.507 104.310   1.776   0.970   0.001   0.030
E E  4NZWB                265    0.402  70.229   1.844   0.923   0.002   0.076
B L  4NZWB                266    0.045   8.294   0.309   0.923   0.002   0.076
B L  4NZWB                267    0.096  17.559   0.519   0.782   0.003   0.216
E K  4NZWB                268    0.676 138.950   1.217   0.600   0.003   0.397
B H  4NZWB                269    0.183  33.360   1.702   0.246   0.004   0.750
E K  4NZWB                270    0.483  99.271   0.814   0.502   0.002   0.495
B F  4NZWB                271    0.150  30.025   0.117   0.717   0.014   0.269
B I  4NZWB                272    0.073  13.579  -0.037   0.751   0.050   0.199
E T  4NZWB                273    0.392  54.384   1.254   0.751   0.050   0.199
E R  4NZWB                274    0.590 135.018   0.946   0.522   0.016   0.462
E Y  4NZWB                275    0.312  66.717   0.404   0.257   0.016   0.727
E T  4NZWB                276    0.426  59.086  -0.391   0.109   0.005   0.886
E K  4NZWB                277    0.682 140.308   0.014   0.016   0.005   0.979
E K  4NZWB                278    0.534 109.926  -0.000   0.058   0.017   0.925
E T  4NZWB                279    0.583  80.848  -0.574   0.257   0.016   0.727
E S  4NZWB                280    0.466  54.592  -0.484   0.339   0.016   0.645
E F  4NZWB                281    0.296  59.367   0.377   0.339   0.016   0.645
B L  4NZWB                282    0.063  11.554   0.070   0.782   0.003   0.216
E T  4NZWB                283    0.471  65.328   1.555   0.923   0.002   0.076
E E  4NZWB                284    0.525  91.717   1.639   0.970   0.001   0.030
B L  4NZWB                285    0.059  10.803   0.969   0.970   0.001   0.030
B I  4NZWB                286    0.067  12.395   0.275   0.970   0.001   0.030
E D  4NZWB                287    0.568  81.806   1.745   0.970   0.001   0.030
E R  4NZWB                288    0.342  78.295   1.723   0.988   0.000   0.012
B Y  4NZWB                289    0.057  12.224   1.029   0.970   0.001   0.030
E K  4NZWB                290    0.409  84.172   1.225   0.970   0.001   0.030
E R  4NZWB                291    0.643 147.201   1.587   0.923   0.002   0.076
B W  4NZWB                292    0.209  50.361   0.359   0.782   0.003   0.216
E K  4NZWB                293    0.598 123.050   0.811   0.502   0.002   0.495
E S  4NZWB                294    0.773  90.549   0.185   0.176   0.004   0.820
E E  4NZWB                295    0.898 156.881   1.210   0.003   0.003   0.994

Explain the output.  Go back.
```

---
